# The Application of Optical Coherence Tomography Angiography in a Patient with Systemic Lupus Erythematosus

**DOI:** 10.7759/cureus.23843

**Published:** 2022-04-05

**Authors:** Ahmed Sameer Alzahrani, Wijdan Alqahtani, Mohammad A Hazzazi, Abdullah S Alqahtani

**Affiliations:** 1 College of Medicine, King Saud Bin Abdulaziz University for Health Sciences College of Medicine, Jeddah, SAU; 2 College of Medicine, King Khalid University, Abha, SAU; 3 Ophthalmology/Surgery, Ministry of National Guard Health Affairs Hospital, Riyadh, SAU; 4 Opthalmogy/Surgery, King Saud Bin Abdulaziz University for Health Sciences College of Medicine, Riyadh, SAU; 5 Opthalmogy/Surgery, King Abdullah International Medical Research Center, Riyadh, SAU; 6 Ophthalmology/Vitreoretinal and Ocular Oncology Surgery, King Saud Bin Abdulaziz University for Health Sciences College of Medicine, Jeddah, SAU; 7 Ophthalmology/Surgery, Ministry of National Guard Health Affairs Hospital, Jeddah, SAU; 8 Ophthalmology/Surgery, King Abdullah International Medical Research Center, Jeddah, SAU

**Keywords:** retina hemorrhage, cotton wool spot, systemic lupus erythromatosus, retinopathy, retinal vasculitis, oct angiography, retina, sle

## Abstract

A 15-year-old girl presented to the emergency department with a history of bilateral blurred vision for one day, with greater severity in the right eye. Fundus examinations revealed cotton wool spots, dot hemorrhage, and hard exudate. She underwent optical coherence tomography angiography (OCTA), which showed the presence of macula ischemia, decreased vascular density, mild retinal fluid, severe ischemia, some macular edema, and vascular sheathing, indicating active vasculitis in the right eye. Systemic lupus erythematosus (SLE) is a chronic autoimmune disease that can affect many organs in the body, including the eye. Ocular involvement is one of the most well-known features. Retinal vasculitis is a rare complication of SLE that is characterized by vascular sheathing that can progress to vaso-occlusion. We report the clinical features of SLE using OCTA.

## Introduction

Systemic lupus erythematosus (SLE) is a chronic autoimmune disease that can affect many organs in the body, including the eye [1]. The diagnosis is made based on laboratory and clinical criteria, and eye involvement does not fall within the criteria [2]. Ocular involvement is one of the most well-known features of SLE, occurring in 30% of patients with SLE and 10% of those with retinopathy. SLE affects every part of the eye, including the eyelids, conjunctiva, episcleral, sclera, cornea, retina, and optic nerve [1,3]. The most common eye manifestation associated with SLE is keratoconjunctivitis, followed by retinopathy. Retinopathy is considered a major vision-threatening condition and patients with this disease have a poor prognosis. The incidence of SLE retinopathy is between 3% and 29% and varies based on population and disease activity [4]. Retinal vasculitis is a rare complication of SLE characterized by vascular sheathing that can progress to vaso-occlusion, which is the final stage of vasculitis. Moreover, the pathophysiology of retinal vasculitis is the activation of the immune system by immune complex deposit [5]. Retinopathy correlates with the activity of SLE, in particular the involvement of the central nervous system (CNS), and it is associated with decreased survival in patients with SLE. Thus, the detection of early changes in the retina is important for preventing and delaying loss of vision [6,7]. Because patients with SLE experience some changes in retinal vascularity due to microangiopathy, fluorescein angiography is used to evaluate the vascularity by using a dye. Fluorescein angiography is an invasive modality and the gold standard for evaluating the retina in patients with SLE, but it has some limitations [8]. On the other hand, optical coherence tomography angiography (OCTA) is a new, noninvasive imaging modality that can assess the microvasculature changes of the retina in patients with SLE without using dye [8]. However, only a few reports have discussed the role of OCTA in patients with SLE. We report the clinical features of SLE using OCTA.

## Case presentation

A 15-year-old girl presented to the emergency department with a history of bilateral blurred vision for one day, with greater severity noted in the right eye. On the second day, she presented with fever, lethargy, and arthralgia, and her initial laboratory results were significant for high creatinine. She was admitted to the hospital for acute kidney injury and further investigation. After hospital admission, the patient was found to be positive for antinuclear antibodies as well as double-stranded DNA antibodies, anti-Smith antibodies, and cardiolipin. Moreover, she had low complement levels of 4 and 3. She was negative for anti-cyclic citrullinated peptides and lupus anticoagulant. After three cultures were obtained, there was no evidence of infection, and all cultures were negative. She was diagnosed with SLE based on clinical features and laboratory findings with the exclusion of drugs, infection, and tumors. The patient had renal impairment due to lupus nephritis based on renal biopsy. She was transferred to the intensive care unit after experiencing a pulmonary hemorrhage based on findings of computed tomography and then shifted to the ward.

**Figure 1:**
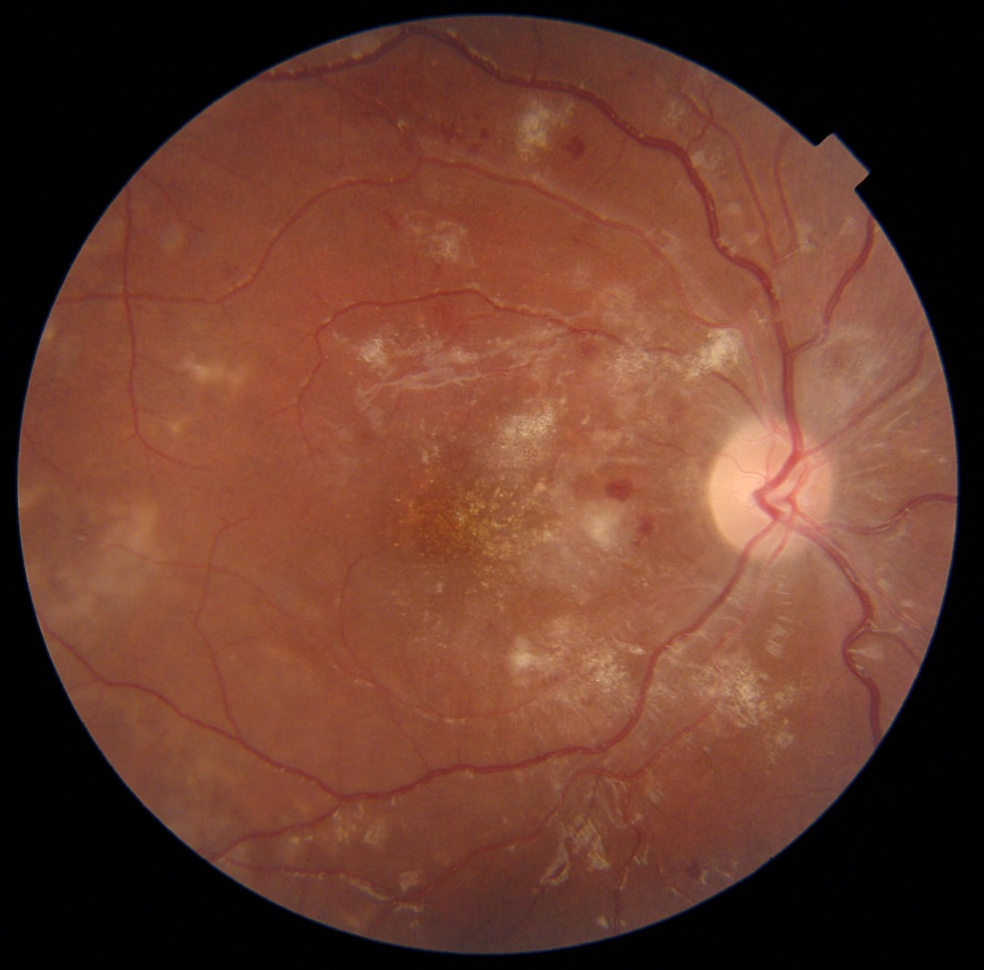
Multiple cotton wool spots in the posterior pole, retinal hemorrhage, some macular edema, and vascular sheathing

During hospital admission, she still complained of blurred vision, which was the same complaint that she had before. Her best-corrected visual acuity was 6/18 and 6/7.5 in the right and left eyes, respectively. Her intraocular pressure was 12 and 12. Anterior chamber examination was normal. Fundus examinations revealed cotton wool spots, dot hemorrhage, and hard exudate. Fluorescein angiography was not practical in this case due to the acute kidney injury resulting from her primary disease. The patient underwent a fundus photo, which revealed the presence of multiple cotton wool spots in the posterior pole, retinal hemorrhage, some macular edema, and vascular sheathing in the right eye (Figure 1).

**Figure 2:**
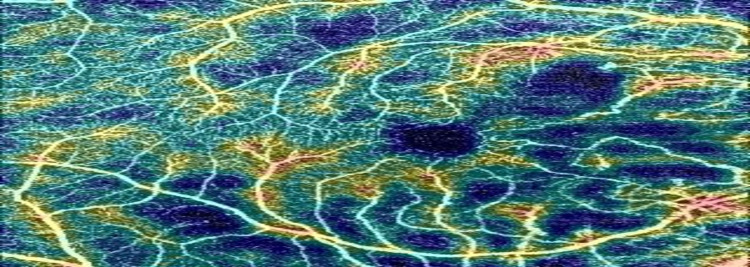
Severe ischemia in the nasal to macula in the right eye.

**Figure 3:**
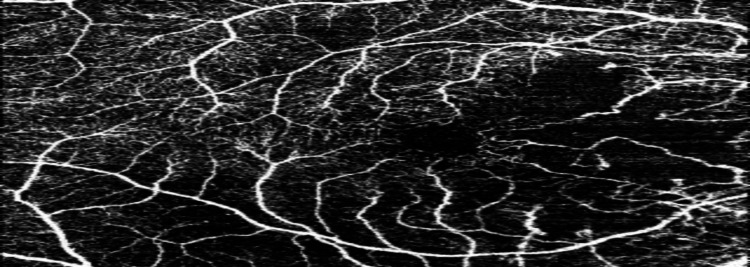
Decreased vascular density.

**Figure 4:**
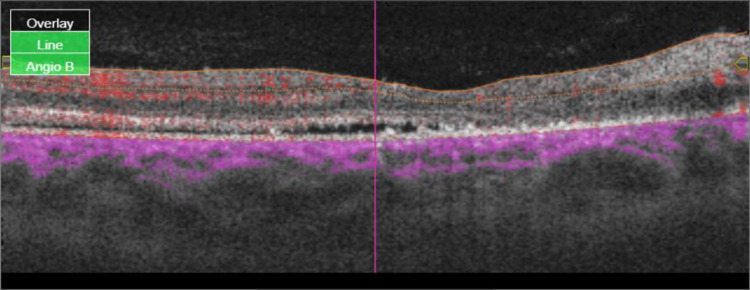
Mild subretinal fluid.

OCTA also showed an area of macula ischemia, decreased vascular density, mild retinal fluid, and severe ischemia in the nasal to macula in the right eye (Figure 2, Figure 3, Figure 4).

**Figure 5:**
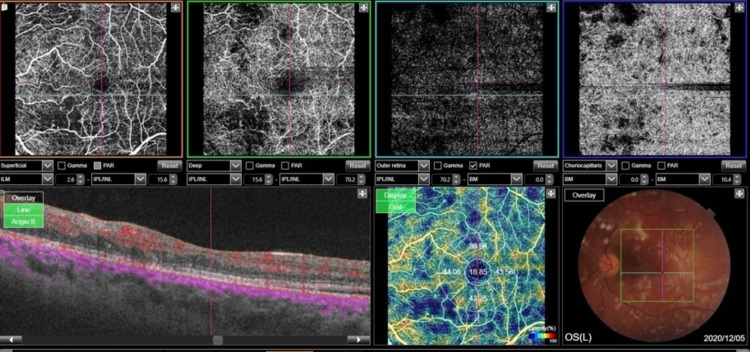
(A, B) Some zones of ischemia in the posterior pole; (C) No subretinal fluid; (D) Multiple cotton wool spots in the posterior pole, retinal hemorrhage, and some macular edema.

OCTA of the left eye showed multiple cotton wool spots in the posterior pole, retinal hemorrhage, and some macular edema but no subretinal fluid (Figure 5).

**Figure 6:**
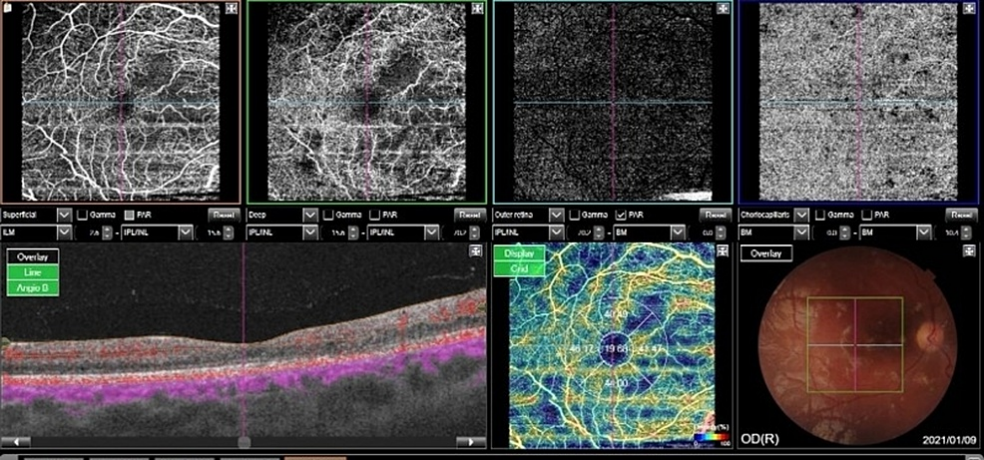
(A, B) Improved ischemia in the nasal to macula in the right eye; (C) Improved subretinal fluid; (D) Improved cotton wool spots in the posterior pole and retinal hemorrhage.

A diagnosis of retinal vasculitis was made. The patient was started on steroid, hydroxychloroquine, anticoagulation, and cyclophosphamide during hospital admission. She was discharged on steroid, hydroxychloroquine, and cyclophosphamide with close follow-up by an ophthalmologist and rheumatologist. After two months, her visual acuity was 6/7.5 in both eyes. A significant improvement was noted in cotton wool spots, hemorrhage, hard exudate, vascularity, and retinal condition (Figure 6).

At the latest follow-up, her visual acuity was 6/7.5 in both eyes, and her OCTA showed improvement in vascularity and retinal condition. 

## Discussion

SLE is an inflammatory autoimmune illness that can affect all systems, including the visual system. The incidence of SLE is higher in females than in males, with a ratio of 9:1 [1]. SLE includes dermatologic, pulmonary, cardiac, renal, musculoskeletal, and eye manifestations [2].

Retinopathy is less commonly seen in patients with SLE, occurring in only 10% of those with eye involvement. Patients with SLE with very active disease are at risk of having retinopathy as compared with those with well-controlled disease (29% vs 3%, respectively) [6]. Findings are similar to diabetic and hypertensive retinopathy, but the exact mechanism of the immune complex deposit is different. The most common finding of SLE retinopathy is cotton wool spots, which represent microinfarctions. Other findings include microaneurysm, dot hemorrhage, and hard exudate [6].

Retinal vasculitis is a rare complication that is considered a subclassification of retinopathy and is characterized by vascular sheathing that occurs in the arterioles or venules. The pathophysiology is an immune complex deposit, which activates the complement system, leading to increased inflammation [5]. A previous study indicated that of patients with SLE with retinal vasculitis, 7% have antiphospholipid syndrome and a high titer of antiphospholipid antibodies, whereas 23% of patients with SLE without retinal vasculitis have high titers of antiphospholipid antibodies [7].

Vaso-occlusion is the final stage of vasculitis and is considered the most severe form of SLE retinopathy. Patients with vaso-occlusion can present with major artery occlusion to microembolisms. Moreover, they can present with more severe features, such as widespread retinal capillary nonperfusion, tractional retinal detachment, vitreous hemorrhage, or central retinal artery or vein occlusion [6].

Fluorescein angiography is an invasive modality that is considered the gold standard of retinal evaluation in patients with SLE. However, it has some disadvantages, such as anaphylactic reaction, acute kidney injury, and its time-consuming nature. Patients with SLE are at risk of chronic kidney diseases, which is a contraindication of fluorescein angiography. A major capillary network such as the superficial and deep capillaries cannot be assessed using fluorescein angiography [8].

OCTA is a new, noninvasive imaging modality that can be used to detect microvasculature changes in the retina of patients with SLE [8]. Moreover, it is used to evaluate the nonperfused or low-perfused areas. One study showed that patients with SLE who underwent OCTA had reduced retinal microvascular density compared with normal eyes. SLE patients with lupus nephritis had reduced retinal microvascular density compared with patients with SLE without lupus nephritis. Most patients with SLE use hydroxychloroquine, which is another risk factor for retinopathy; however, hydroxychloroquine has been found to be one of the protective factors that may preserve vascularity [9]. OCTA, as a component of screening, can also be used to assess preclinical changes in the retina of patients with SLE. One study suggested that patients with SLE with asymptomatic retinopathy based on OCTA should receive regular follow-up as they are at risk of CNS diseases [8].

## Conclusions

OCTA is a new noninvasive imaging modality that can assess the microvasculature changes of the retina in SLE patients without a dye. It can be used as an assessment modality for SLE patients who have acute kidney injuries.
